# The serum thioredoxin-1 levels are not associated with bronchopulmonary dysplasia and retinopathy of prematurity

**DOI:** 10.1038/s41390-024-03078-7

**Published:** 2024-02-16

**Authors:** Mitsuhiro Haga, Nobuhiko Nagano, Junichi Ozawa, Kosuke Tanaka, Naoyuki Miyahara, Takeshi Fujimoto, Kuniya Ishii, Fumihiko Namba

**Affiliations:** 1 Department of Pediatrics, Saitama Medical Center, Saitama Medical University, Kawagoe, Saitama Japan; 2 Department of Pediatrics and Child Health, Nihon University School of Medicine, Tokyo, Japan; 3 Division of Neonatology, Department of Pediatrics, Heersink School of Medicine, The University of Alabama at Birmingham, Birmingham, AL USA

## Abstract

**Background:**

We hypothesized that the serum TRX-1 in extremely preterm infants (EPIs) after birth was associated with the development of severe bronchopulmonary dysplasia (BPD) and retinopathy of prematurity (ROP).

**Methods:**

This single-centered retrospective study enrolled EPIs treated at our institution. Serum TRX-1 concentrations of the residual samples taken on admission, day 10–20 of life, and 36–40 weeks of postmenstrual age (PMA) were measured with an enzyme-linked immunosorbent assay.

**Results:**

The serum TRX-1 levels on admission were not different between the severe BPD (*n* = 46) and non-severe BPD groups (*n* = 67): [median (interquartile range) 147 (73.0–231) vs. 164 (80.5–248) ng/mL] (*P* = 0.57). These had no significant difference between the severe ROP (*n* = 47) and non-severe ROP groups (*n* = 66): [164 (71.3–237) vs. 150 (80.9–250) ng/mL] (*P* = 0.93). The TRX-1 levels at 10–20 days of life and 36–40 weeks of PMA also had no association with the development of severe BPD and ROP.

**Conclusion:**

The serum TRX-1 levels after birth are not predictive of severe BPD and ROP.

**Impact:**

- Serum thioredoxin-1 levels in extremely preterm infants on the day of birth are lower than those in term or near-term infants hospitalized for transient tachypnea of the newborn.
- In extremely preterm infants, the serum thioredoxin-1 levels on the day of birth, at 10–20 days of life, and at postmenstrual age of 36–40 weeks were not associated with severe bronchopulmonary dysplasia and retinopathy of prematurity.
- The thioredoxin system is under development in extremely preterm infants; however, the serum thioredoxin-1 level is not predictive for severe bronchopulmonary dysplasia and retinopathy of prematurity.

## Introduction

The intrauterine environment for fetal growth and development contains a lower oxygen concentration than the atmospheric level; in the second trimester, oxygen partial pressure in the umbilical vein is reported to be around 30–55 mmHg.^1,2^ All neonates experience a drastic increase in oxygen exposure at birth. While oxygen is essential for energy production, it can induce damage to proteins, lipids, and deoxyribonucleic acid (DNA) by forming reactive oxygen species (ROS).^3^ Several antioxidant enzymes, such as superoxide dismutase and catalase, protect the human body from these oxidative injuries.^4^ Preterm neonates are more susceptible to oxidative stress than term neonates due to their organ immaturity and limited capacity for synthesizing antioxidant enzymes.^5^ The tissue injury from ROS is considered to be associated with some complications from preterm birth, such as bronchopulmonary dysplasia (BPD) and retinopathy of prematurity (ROP).^6^

The thioredoxin system, including thioredoxin, thioredoxin reductase, and nicotinamide adenine dinucleotide phosphate, is one of the major antioxidant systems functioning in the human body.^7^ Thioredoxin is a small protein (molecular mass: 12 kDa) with a redox-active site of -Cys-Gly-Pro-Cys-.^8^ Thioredoxin-1 (TRX-1) is one of the three isoforms of thioredoxin, which is mainly located in the cytosol and is released into the extracellular space in response to oxidative stress.^8^ TRX-1 plays a wide range of roles in the human body: scavenging ROS, reducing inflammation, synthesizing DNA, regulating gene expression, and controlling cell apoptosis.^9,10^ The serum/plasma TRX-1 levels are predictive of various diseases, such as chronic obstructive pulmonary disease, acute or chronic heart failure, acute ischemic stroke, infectious diseases, and malignant tumors.^9,10^ In healthy adults, the reference range of the serum TRX-1 is 10–30 ng/mL, but these can rise to 40–140 ng/mL in the aforementioned conditions.^8^ However, few clinical studies investigated the role of TRX-1 in newborn diseases, and there is no established reference range of the serum TRX-1 levels in neonates. In animal studies, overexpression of TRX attenuates the severity of hyperoxic-induced lung injury^11^ and retinopathy^12^ by suppressing proinflammatory cytokines in neonatal mice models, suggesting that higher TRX expression is protective against oxygen-induced inflammation in newborn animals.

The primary objective of this study was to investigate the relationship between serum TRX-1 levels after birth and the development of severe BPD and severe ROP in extremely preterm infants (EPIs, born at 22–27 weeks of gestational age [GA]). TRX-1 was hypothesized to protect against the pathogenesis of BPD and ROP, and the serum TRX-1 levels could be a predictive factor for these complications in EPIs. The secondary objective was to investigate the developmental aspect of the TRX-1 system by comparing the serum TRX-1 levels of EPIs and term or near-term neonates on the day of birth. It was hypothesized that the thioredoxin system is under development in EPIs, and their serum TRX-1 levels on the day of birth would be lower than those of term or near-term infants.

## Methods

### Study design

This single-centered retrospective cohort study enrolled EPIs who were either born at or transferred to Saitama Medical Center, Saitama Medical University, Kawagoe, Japan, from August 2015 to May 2021. The exclusion criteria were infants with major congenital abnormalities or who died before 36 weeks of postmenstrual age (PMA). Additionally, a control group was selected, which consisted of neonates born at 36–40 weeks of GA and hospitalized at our institution with the diagnosis of transient tachypnea of the newborn (TTN). Clinical data of relevant patients were identified and extracted from the institution’s medical records. We measured TRX-1 concentrations of the residual serum samples of EPIs obtained on the day of birth, at 10–20 days of life, and 36–40 weeks of PMA. The TRX-1 levels of the control group on the day of birth were also measured. Reference ranges of TRX-1 concentration in each time point of EPIs and that of the control group on the day of birth were established. We analyzed the difference between the serum TRX-1 levels on the day of birth in the EPI group and the control group. Next, we compared TRX-1 levels of the three points after birth between the patients who developed severe BPD and those who did not. In the same way, TRX-1 levels between the patients who developed severe ROP and those who did not were also compared.

Patient consent was not required. Parents are given information and the opportunity to opt out from data collection for this study on our institution’s website. The study protocol was approved by the Ethics Committee for Clinical Research of Saitama Medical Center, Saitama Medical University (approval number: 2351-II).

### Sample size estimation

The sample size was calculated using the web-based program of PS: Power and Sample Size Calculation® version 3.1.6.^13^ The calculation was performed with an assumption of a two-paired t-test with an estimated standard deviation (SD) of 12 ng/mL from the previously reported serum TRX concentration in umbilical cord blood.^14^ The significance and power were determined as 0.05 and 0.80, respectively. The required minimum number of patients in each independent group was calculated as 24 to detect 10 ng/mL of the difference in TRX levels. Because our institution’s prevalence of severe BPD and severe ROP in the period of 2015–2018 was 25 and 35%, respectively, we set the target sample size of EPIs as 96.

### Definitions

Hyperglycemia in pregnancy included gestational diabetes mellitus, overt diabetes in pregnancy, and pregestational diabetes mellitus; those diagnoses were based on the Japanese diagnostic criteria.^15,16^ Hypertensive disorders of pregnancy (HDP) were defined based on the current version of Japanese criteria.^17^ Placental histology was assessed by pathologists in our institution, and histologic chorioamnionitis was defined as stage 2 and 3 chorioamnionitis according to the criteria reported by Blanc.^18^ Antenatal corticosteroid therapy (ACS) was defined as any corticosteroid administration before delivery to enhance fetus maturation. Magnesium sulfate (MgSO_4_) administration was defined as the intravenous MgSO_4_ infusion before delivery for any purpose. Ritodrine hydrochloride, a short-acting β2 adrenoreceptor agonist widely used for tocolysis in Japan, is defined as a continuous intravenous infusion of that agent before delivery.^19^ Small-for-gestational-age (SGA) was defined as infants whose both birth weight (BW) and height were <10th percentile of the Japanese neonatal anthropometric charts for GA at birth.^20^ Respiratory distress syndrome was diagnosed by experienced neonatologists based on the findings of the clinical course, stable microbubble test, and chest x-ray.^21^ Persistent pulmonary hypertension of the newborn was defined as pulmonary hypertension in the acute phase after birth requiring nitric oxide inhalation therapy. Length of mechanical ventilation was defined as the duration of invasive ventilation before patients were successfully weaned off from ventilators. We did not include the period of mechanical ventilation for medical procedures, such as ophthalmology tests or surgery with general anesthesia after the patients were successfully weaned off from the ventilators. BPD was defined as the requirement of supplemental oxygen or positive pressure support on the 28th day after birth. The severity of BPD was classified according to the *Eunice Kenndy Shriver* National Institute of Child Health and Human Development criteria: mild, breathing room air at 36 weeks PMA; moderate, need for <30% oxygen at 36 weeks PMA; severe, need for ≥30% oxygen and/or positive pressure at 36 weeks PMA.^22^ The diagnosis and grading of ROP were made by experienced ophthalmologist in our institution based on the international classification.^23^ Severe ROP was defined as ROP requiring any treatment (laser photocoagulation or vitreous surgery) or stages 4 and 5 ROP. TTN was defined as respiratory distress after birth requiring any respiratory support without evidence of other known diseases.

### TRX measurements

Venous or arterial blood samples were collected in plain tubes on the day of birth, at 10–20 days of life, and 36–40 weeks of PMA. The samples were immediately centrifuged and stored at −80 °C in our facility until the TRX measurements. The samples were thawed at room temperature and diluted ten times with normal saline. TRX concentrations were duplicately measured using Human Thioredoxin Assay Kit^®^ (Immuno-Biological Laboratories, Fujioka, Japan). An average of the results from the duplicated sample were determined as each patient’s serum TRX concentration.

### Statistical analyses

The Fischer’s exact test and Mann–Whitney U test were used to assess the between-group differences in clinical characteristics for nominal and continuous variables, respectively. In a preliminary analysis, both TRX-1 and log-transformed TRX-1 values of the EPI group did not conform to a normal distribution as assessed using the Shapiro–Wilk test (*P* < 0.05). Hence, we regarded TRX-1 as a non-parametric variable for this study; accordingly, the Mann–Whitney U test was used to assess the between-group difference in TRX-1 levels. The median and 95% range of TRX-1 levels in the EPI and control groups were determined by the smoothed empirical likelihood method. The Wilcoxon signed-rank test was used to analyze changes in the serum TRX-1 levels of EPIs from birth to 10–20 days of life and to 36–40 weeks of PMA. Adjusted odds ratios (aOR) and 95% confidence intervals of TRX-1 for severe BPD and severe ROP were calculated using multivariate logistic regression analyses simultaneously adjusted for GA, BW, and ACS as known predictors. The existence of multicollinearity was assessed with the variance inflation factors for independent variables, and the Wald test was used to determine the significance of the predictors in the logistic regression models. Missing data were not imputed. We considered a two-tailed *P* value < 0.05 as statistically significant. Statistical analyses were performed using JMP® Version 16.0 (SAS Institute Inc., Cary, NC).

## Results

### EPI group

**Fig. 1:**
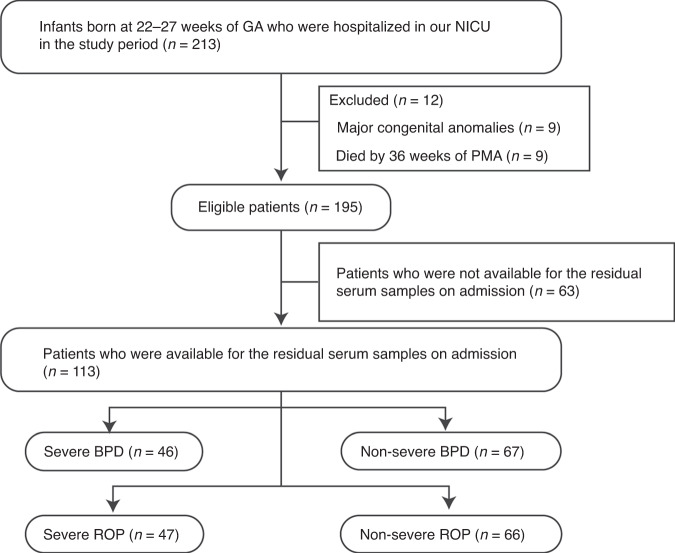
Flow diagram presenting the number of patients at each step in the study. GA gestational age, NICU neonatal intensive care unit, PMA postmenstrual age, BPD bronchopulmonary dysplasia, ROP retinopathy of prematurity.

In this study period, 213 EPIs were hospitalized at our institution. We excluded infants with major congenital anomalies (*n* = 9) and those who died within 36 weeks of PMA (*n* = 9) from this study. Out of the remaining 195 infants, the residual serum samples on admission were available in 113 (58%) patients, and they were subjected to subsequent TRX-1 measurements and analyses (Fig. 1). The patients who developed severe BPD and severe ROP were 46/113 (41%) and 47/113 (42%), respectively. Out of 113 patients, residual serum samples at 10–20 days and at 36–40 weeks of PMA were available in 47 (42%) and 70 (62%), respectively.

### Control group

This group comprised 32 infants born at >36 weeks of GA and >1800 g of BW hospitalized for TTN at our institution between November 2017 and June 2020.

### Comparison of serum TRX-1 levels between the EPI and control groups on admission

Table 1 presents a comparison of clinical characteristics between the EPI and control group infants. Except for one infant with Hispanic ethnicity, the maternal ethnicity for all infants was Asian. The frequencies of ACS, MgSO_4_, and ritodrine hydrochloride administration were higher in the EPI group than in the control group (*P* < 0.001 each).

**Table 1:** Comparison of clinical characteristics and serum thioredoxin-1 levels between extremely preterm infants and control patients.

Characteristics^a^	EPIs (*n* = 113)	Control (*n* = 32)	*P* value^b^
Maternal ethnicity			1.0
Asian	112/113 (99)	32/32 (100)	
Hispanic	1/113 (1)	0/60	
Maternal age	33 (30–37)	34 (29–37)	0.90
Primiparity	55/112 (49)	14/32 (44)	0.69
Multiple pregnancy	18/113 (16)	6/32 (19)	0.79
HDP	15/113 (13)	1/32 (8.3)	0.20
Hyperglycemia in pregnancy	7/113 (6.2)	3/32 (9.4)	0.69
Antenatal corticosteroids	58/113 (51)	0/32	<0.001
MgSO_4_	61/113 (54)	1/32 (3.1)	<0.001
Ritodrine hydrochloride	92/113 (81)	4/32 (13)	<0.001
Cesarean section	91/113 (81)	23/32 (72)	0.33
Male sex	56/113 (50)	16/32 (50)	1.0
Gestational age (weeks)	25.6 (23.9–26.6)	37.3 (36.6–39.0)	<0.001
Birthweight (g)	669 (561–884)	2804 (2481–3122)	<0.001
SGA	26/113 (23)	2/32 (6.3)	0.04
Neonatal transfer	3/113 (2.7)	5/60 (8.3)	0.13
Apgar score (1 min.)	4 (2–5)	8 (6–8)	<0.001
Apgar score (5 min.)	7 (5–8)	8 (8–9)	<0.001
TRX-1 (ng/mL)			
on admission	153 (74.5–242)	267 (150–428)	<0.001
at 10–20 days of life	*n* = *47* 111 (65.1–234)		
at 36–40 weeks of PMA	*n* = *70* 33.6 (18.2–87.2)		

*TRX-1* thioredoxin-1, *HDP* hypertensive disorders of pregnancy, *MgSO*_*4*_ magnesium sulfate administration, *SGA* small-for-gestational-age.

^a^Data are presented as a number/denominator (percentage) for nominal variables or median (interquartile range) for continuous variables. Note that denominators differ in each variable due to data availability.

^b^P values for the differences in nominal and continuous variables between the two groups were determined by the exact Fischer test and Mann–Whitney U test, respectively.

**Fig. 2:**
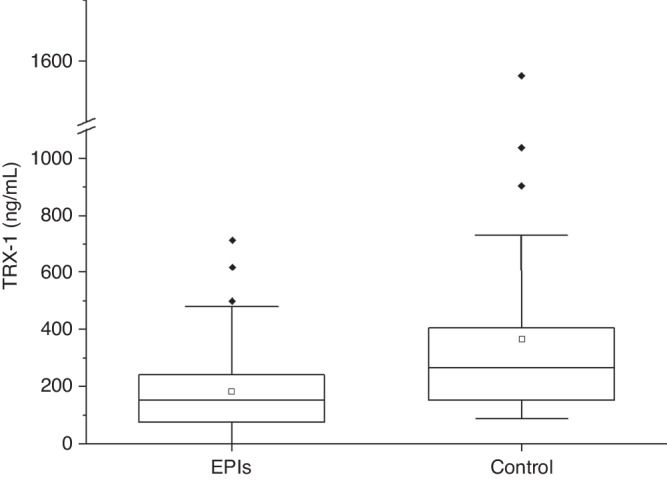
Box-whisker plots of thioredoxin-1 (TRX-1) in the extremely preterm infants (EPIs) and control groups. The median (interquartile range) of TRX levels on admission in the EPIs group was 153 (74.5–242) ng/mL, and that in the control group was 267 (150–428) ng/mL. The TRX-1 of the EPIs group was significantly lower than that of the control group (*P* < 0.001). EPIs extremely preterm infants, TRX-1 thioredoxin-1.

**Fig. 3:**
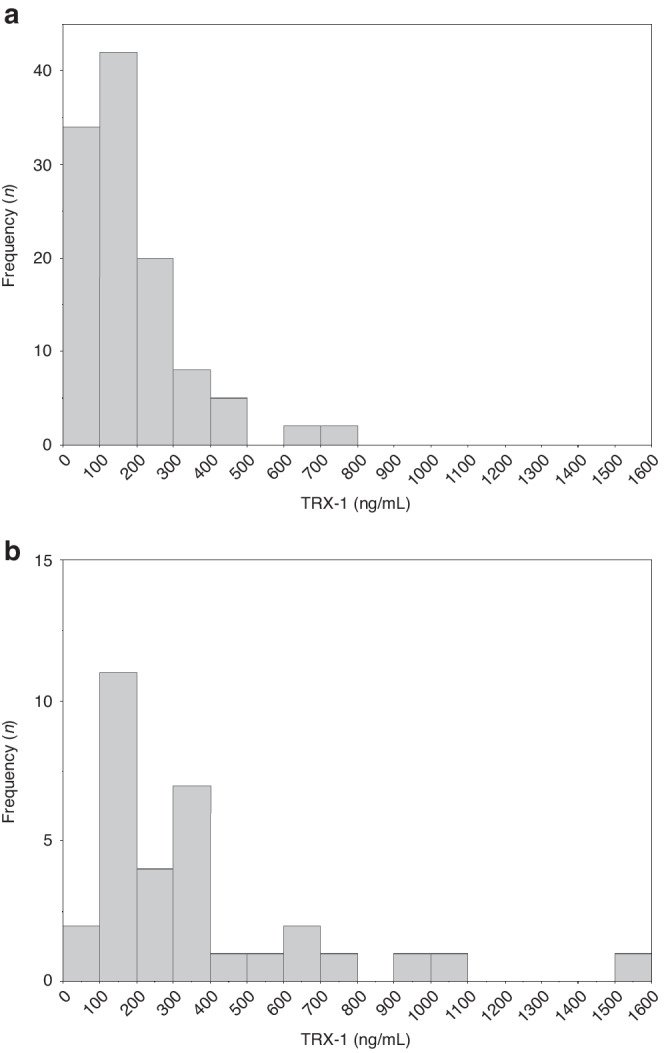
Histograms of the serum thioredoxin-1 levels at the day of birth Histograms of the serum thioredoxin-1 levels in extremely preterm infants (**a**) and controls (**b**). Both histograms show the distributions of TRX-1 skewed to the right. TRX-1 thioredoxin-1.

**Fig. 4:**
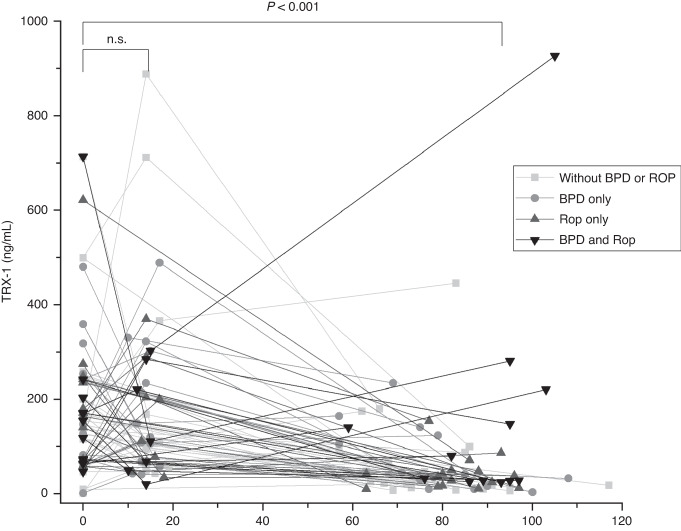
Changes of the serum thioredoxin-1 levels after birth. Thioredoxin-1 levels were measured in the residual serum samples taken on the day of birth, at 10–20 days of birth, and at 36–40 weeks of postmenstrual age. Square, round, triangle, and inverted triangle symbols indicate the patients without severe BPD or ROP, those with severe BPD only, those with severe ROP only, and those with severe BPD and ROP. Each patient’s value is connected with a black line. Median (interquartile range) of the serum TRX-1 levels on the day of birth, at 10–20 days of life, and 36–40 weeks of PMA was 153 (74.5–242), 111 (65.1–234), and 33.6 (18.2–87.2) ng/mL, respectively. The Wilcoxon signed-rank test showed a significant difference between on the day of birth and at 36–40 weeks of PMA (*P* < 0.001). No significant difference was shown between on the day of birth and at 10–20 days of life. TRX-1 thioredoxin-1.

The median [interquartile range (IQR)] TRX-1 level in the EPI group on admission was 153 (74.5–242) mg/mL (range = 1.07–714 ng/mL), and that in the control group was 267 (150–428) ng/mL (range = 86.8–1576 ng/mL). The TRX-1 levels on admission in the EPI group were significantly lower than that in the control group (*P* < 0.001) (Table 1 and Fig. 2). The histograms of the serum TRX-1 were skewed to the right (Fig. 3), but the histograms of logit-transformed TRX-1 levels showed almost symmetrical distribution (Supplementary Fig. S1). In the EPI group, The TRX-1 levels were 111 (65.1–234) ng/mL at 10–20 days of life and 33.6 (18.2–87.2) ng/mL at 36–40 weeks of PMA (Table 1). Compared to that upon admission, the TRX-1 levels at 10–20 days of life were not significantly different (*P* = 0.88), but these were significantly lower at 36–40 weeks of PMA (*P* < 0.001) (Fig. 4). The estimated values of the median and 95% range of serum TRX-1 in the EPI and control groups are shown in Table 2.

**Table 2:** The estimated median value and 95% range for serum thioredoxin-1 levels (ng/mL).

	Median	2.5% Lower Limit	97.5% Higher Limit
EPI			
on admission (*n* = 113)	152 (134–170)	14.4 (0.00–32.9)	625 (484–716)
on day10–20 of life (*n* = 47)	114 (89.3–165)	10.7 (0.00–21.0)	738 (379–907)
at 36–40 weeks of PMA (*n* = 70)	34.7 (28.0–43.5)	0.00 (0.00–1.68)	440 (196–930)
Control			
on admission (*n* = 32)	263 (200–342)	50.1 (27.8–75.1)	1529 (788–1627)

*CI* confidence interval, *EPI* extremely preterm infant.

### Comparison of serum TRX-1 levels between the severe and non-severe BPD groups

GA, BW, and Apgar scores at 5 min were lower in the severe BPD group than in the non-severe BPD group (Table 3). The median (IQR) TRX-1 concentrations on admission, at 10–20 days of life, and at 36–40 weeks of PMA were 147 (73.0–231), 120 (62.6–294), and 34.7 (26.0–141) ng/mL, respectively, in the severe BPD group. The corresponding TRX-1 levels in the non-severe BPD group were 164 (80.5–248), 105 (64.1–201), and 29.1 (17.4–49.9) ng/mL, respectively (Table 3). No statistically significant difference between the two groups was observed in the TRX-1 levels of each point (*P* = 0.57).

**Table 3:** Comparison of clinical characteristics between the severe bronchopulmonary dysplasia (BPD) and non-severe BPD groups.

Characteristics^a^	Severe BPD (*n* = 46)	Non-severe BPD (*n* = 67)	*P* value^b^
Maternal ethnicity			1.0
Asian	46/46 (100)	66/67 (99)	
Hispanic	0/46	1/67 (1.5)	
Maternal age	34 (30–36)	33 (29–37)	0.97
Primiparity	21/46 (46)	34/67 (51)	0.70
Multiple pregnancy	6/46 (13)	12/67 (18)	0.60
HDP	8/46 (17)	7/67 (10)	0.40
Hyperglycemia in pregnancy	5/46 (11)	2/67 (3)	0.12
Antenatal corticosteroids	27/46 (59)	31/67 (46)	0.25
MgSO_4_	25/46 (54)	36/67 (54)	1.0
Ritodrine hydrochloride	39/46 (85)	53/67 (79)	0.47
CAM, histological^c^	21/45 (47)	35/66 (53)	0.56
Cesarean section	41/46 (89)	50/67 (75)	0.09
Male sex	26/46 (57)	30/67 (45)	0.25
Gestational age (weeks)	24.7 (23.4–26.3)	25.7 (24.3–26.9)	0.03
Birthweight (g)	598 (496–796)	778 (607–921)	0.002
SGA	14/46 (30)	12/67 (18)	0.17
Apgar score (1 min.)	3 (1–5)	6 (5–7)	0.07
Apgar score (5 min.)	4 (3–5)	7 (6–8)	<0.001
Neonatal transfer	1/46 (2)	2/67 (3)	1.0
RDS	42/46 (91)	53/67 (79)	0.12
Air leak syndrome	3/46 (7)	2/67 (3)	0.40
PPHN	9/46 (20)	7/67 (10)	0.18
Mechanical ventilation (days)	52 (40–64)	30 (15–46)	<0.001
PMA at extubation (weeks)	31.9 (30.8–34.0)	30.0 (28.9–31.6)	<0.001
PDA clipping	8/46 (17)	5/67 (7)	0.14
IVH (any grade)	14/46 (30)	16/67 (24)	0.52
Severe IVH (grades 3 and 4)	5/46 (11)	3/67 (4.5)	0.27
PVL, cystic	1/46 (2.2)	0/67	0.41
NEC	2/46 (4.4)	0/67	0.16
FIP	2/46 (4.4)	5/67 (7.5)	0.70
Sepsis	8/46 (17)	8/67 (12)	0.43
Severe ROP	21/46 (46)	26/67 (39)	0.56
TRX-1 (ng/mL)			
on admission	*n* = *46* 147 (73.0–231)	*n* = *67* 164 (80.5–248)	0.57
on day 10–20	*n* = *21* 120 (62.6–294)	*n* = *26* 105 (64.1–201)	0.57
at 36–40 weeks of PMA	*n* = *27* 34.7 (26.0–141)	*n* = *43* 29.1 (17.4–49.9)	0.16

*BPD* bronchopulmonary dysplasia, *CAM* chorioamnionitis, *FIP* focal intestinal perforation, *HDP* hypertensive disorders of pregnancy, *IVH* intra-ventricular hemorrhage, *MgSO*_*4*_ magnesium sulfate administration, *NEC* necrotizing enterocolitis, *PDA* patent ductus arteriosus, *PMA* postmenstrual age, *PPHN* persistent pulmonary hypertension of the newborn, *PVL* periventricular leukomalacia, *RDS* respiratory distress syndrome, *ROP* retinopathy of prematurity, *SGA* small-for-gestational-age, *TRX-1* thioredoxin-1.

^a^Data are presented as a number/denominator (percentage) for nominal variables or median (interquartile range) for continuous variables. Note that denominators differ in each variable due to data availability.

^b^P values for the differences of nominal and continuous variables between the two groups were determined by the exact Fischer test and Mann–Whitney U test, respectively.

^c^Data are missing in one patient in the severe BPD group and one patient in the non-severe BPD group.

### Comparison of serum TRX-1 levels between the severe and non-severe ROP groups

A comparison of the severe and non-severe ROP groups revealed that the frequencies of PDA clipping and IVH were higher in the former than in the latter (Table 4). The number of days of mechanical ventilation was also greater in the severe ROP group. The median (IQR) TRX-1 concentration on admission, at 10–20 days of life, and at 36–40 weeks of PMA in the severe ROP group were 164 (71.3–237), 111 (53.8–269), and 37.1 (25.0–85.3) ng/mL, respectively. The corresponding TRX levels in the non-severe ROP group were 150 (80.9–250), 108 (65.1–234), and 31.2 (15.9–90.2) ng/mL. No statistically significant difference between the two groups was observed in TRX-1 levels after birth (Table 4).

**Table 4:** Comparison of clinical characteristics between the severe retinopathy of prematurity (ROP) and non-severe ROP groups.

Characteristics^a^	Severe ROP (*n* = 47)	Non-severe ROP (*n* = 66)	*P* value^b^
Maternal ethnicity			1.0
Asian	47/47 (100)	65/66 (98)	
Hispanic	0/47	1/66 (1.5)	
Maternal age	34 (31–37)	33 (30–36)	0.41
Primiparity	26/47 (55)	29/66 (44)	0.26
Multiple pregnancy	9/47 (19)	9/66 (14)	0.45
HDP	3/47 (6.4)	12/66 (18)	0.09
Glycemic disorders in pregnancy	4/47 (8.5)	3/66 (4.6)	0.45
Antenatal corticosteroids	22/47 (47)	36/66 (55)	0.45
MgSO_4_	23/47 (49)	38/66 (58)	0.44
Ritodrine hydrochloride	41/47 (87)	51/66 (77)	0.22
CAM, histological^c^	20/46 (43)	36/65 (55)	0.25
Cesarean section	40/47 (85)	51/66 (77)	0.34
Male sex	24/47 (51)	32/66 (48)	0.85
Gestational age (weeks)	24.9 (23.6–26.3)	25.9 (24.3–26.9)	0.04
Birthweight (g)	666 (539–807)	747 (565–942)	0.09
SGA	12/47 (26)	14/66 (21)	0.65
Apgar score (1 min.)	3 (2–5)	4 (3–5)	0.22
Apgar score (5 min.)	6 (5–7)	7 (6–8)	0.12
Neonatal transfer	1/47 (2.1)	2/66 (3.0)	1.0
RDS	41/47 (87)	54/66 (82)	0.60
Air leak syndrome	4/47 (8.59	1/66 (1.5)	0.16
PPHN	6/47 (13)	10/66 (15)	0.79
Mechanical ventilation (days)	46 (33–58)	38 (15–53)	0.03
PMA at extubation (weeks)	31.1 (29.6–32.9)	30.6 (28.9–32.1)	0.17
Severe BPD	21/47 (45)	25/66 (38)	0.56
PDA clipping	9/47 (19)	4/66 (6.1)	0.04
IVH (any grade)	18/47 (38)	12/66 (18)	0.03
Severe IVH (grades 3 and 4)	3/47 (6.4)	5/66 (7.6)	1.0
PVL, cystic	1/47 (2.1)	0/66	0.42
NEC	0/47	2/66 (3.0)	0.51
FIP	4/47 (8.5)	3/66 (4.6)	0.45
Sepsis	9/47 (19)	7/66 (11)	0.27
TRX-1 (ng/mL)			
on admission	*n* = *47* 164 (71.3–237)	*n* = *66* 150 (80.9–250)	0.93
on day 10–20	*n* = *16* 111 (53.8–269)	*n* = *31* 108 (65.1–234)	0.16
at 36–40 weeks of PMA	*n* = *28* 37.1 (25.0–85.3)	*n* = *42* 31.2 (15.9–90.2)	0.26

*BPD* bronchopulmonary dysplasia, *CAM* chorioamnionitis, *FIP* focal intestinal perforation, *HDP* hypertensive disorders of pregnancy, *IVH* intra-ventricular hemorrhage, *MgSO*_*4*_ magnesium sulfate administration, *NEC* necrotizing enterocolitis, *PDA* patent ductus arteriosus, *PMA* postmenstrual age, *PPHN* persistent pulmonary hypertension of the newborn, *PVL* periventricular leukomalacia, *RDS* respiratory distress syndrome, *ROP* retinopathy of prematurity, *SGA* small-for-gestational-age, *TRX-1* thioredoxin-1.

^a^Data are presented as a number/denominator (percentage) for nominal variables or median (interquartile range) for continuous variables. Note that denominators differ in each variable due to data availability.

^b^P values for the differences in nominal and continuous variables between the two groups were determined by the exact Fischer test and Mann–Whitney U test, respectively.

^c^Data are missing in one patient in the severe ROP group and one patient in the non-severe ROP group.

### Multivariate logistic regression analyses for severe BPD and ROP

Table 5 presents the results of multivariate logistic regression models for severe BPD and severe ROP using GA, BW, ACS, and TRX-1 on admission as predictors. We found no evidence for multicollinearity (variance inflation factors for the independent variables <2.0 in every model). In the models, TRX-1 levels on admission have no significant independent effect on severe BPD (aOR 1.0, 95% CI 0.97–1.0) and severe ROP (aOR 1.0, 95% CI 0.98–1.0). Similar results were obtained in the multivariate logistic regression models with the predictor of TRX-1 at different time points. (Supplementary Tables S1 and S2).

**Table 5:** Multivariate logistic regression analyses for severe bronchopulmonary dysplasia and severe retinopathy of prematurity.

	Severe BPD^a^					Severe ROP^b^				
Variables	B	SE	Wald χ^2^	*P*^c^	aOR (95% CI)	B	SE	Wald χ^2^	*P*^c^	aOR (95% CI)
Gestational age (weeks)	−0.16	0.16	1.0	0.31	0.85 (0.62–1.2)	−0.16	0.16	1.0	0.31	0.85 (0.62–1.2)
Birth body weight (100 g)	−0.22	0.13	2.9	0.09	0.80 (0.63–1.0)	−0.12	0.12	0.83	0.33	0.89 (0.70–1.1)
Antenatal corticosteroids	0.35	0.21	2.7	0.10	2.0 (0.87–4.7)	−0.11	0.20	0.27	0.61	0.81 (0.36–1.8)
TRX-1 (10 ng/mL)	−0.0030	0.014	0.044	0.83	1.0 (0.97–1.0)	0.0088	0.014	0.40	0.53	1.0 (0.98–1.0)

Area under the receiver operating characteristics curve: ^a^0.69 and ^b^0.62.

*B* coefficient, *SE* standard error, *aOR* adjusted odds ratio, *CI* confidence interval, *TRX-1* thioredoxin-1.

^c^A P value for the aOR of each variable was determined by the Wald test for the multivariate logistic regression model.

## Discussion

To the best of our knowledge, this is the first study measuring the serum TRX-1 levels in neonates. The median (IQR) of the serum TRX-1 levels in the EPI and control groups were 153 (74.5–242) and 267 (150–428) ng/mL, respectively, which are much higher than the reference value in healthy adults of 10–30 ng/mL.^8^ Longitudinal measurements of TRX-1 levels in EPIs revealed that the serum TRX-1 levels remained high at least until 10–20 days of life and thereafter declined to the approximate adult reference value in 36–40 weeks of PMA. This study also revealed that the serum TRX-1 concentration on the day of birth was lower in EPIs than in term or near-term neonates hospitalized for TTN, suggesting that the thioredoxin system is under development in preterm infants like other antioxidant systems. However, our primary hypothesis that the serum TRX-1 levels would be a predictor for the development of severe BPD and severe ROP was not supported; there was no statistically significant relationship between serum TRX-1 levels at any point and the development of severe BPD and ROP in EPIs.

The serum TRX-1 levels on the day of birth were apparently higher in both the EPI and control groups than those of the healthy adult population. TRX-1 production is enhanced in the perinatal period to counter the sudden increase of oxidative stress. The dynamic change in TRX-1 concentration of EPIs after birth is assumed to reflect the clinical course of EPIs. In the first 2–3 weeks of life, they are unstable and require various interventions, but toward term-equivalent age, their condition stabilizes. The serum TRX-1 trend is consistent with the adaptation process to the ex-utero environment in EPIs.

The developmental aspect of the thioredoxin system has not been thoroughly investigated. This study showed that the serum TRX-1 levels on the day of birth in the EPI group were significantly lower than those of neonates born at ≥36 weeks of gestation with TTN. It is consistent with the results of previous animal studies. Demarquoy et al. reported that the enzymes of the thioredoxin system in the rat liver increased in the later fetal period.^24^ However, the previous clinical study by Todoroki et al. showed an inconsistent result; they showed an inverse relationship between GA and thioredoxin concentration in umbilical cord blood.^14^ This apparent difference with our results may be attributable to the difference in the production of thioredoxin system enzymes before and after birth. The distribution of thioredoxin expression in fetuses differs depending on the organs.^25^ The placenta is one of the organs producing thioredoxin, and its expression is higher in preterm pregnancy than in term pregnancy.^26,27^ On the contrary, thioredoxin production in the human lungs is reported to begin after they enter the alveolar stage, 36 weeks of PMA or later.^28^ The study using umbilical cord blood can be affected by the placental production of thioredoxin. We measured the TRX-1 levels using samples from the neonate to assess the potency of production of TRX-1 after exposure to an ex-utero environment. We selected TTN patients as controls because their lungs experience oxidative stress from oxygen supplementation or ventilator support, similar to most EPIs. We found that TRX concentration was lower in the EPI group, and this corroborated the findings of the animal study conducted by Das et al.^29^ They revealed that the activities of thioredoxin and thioredoxin reductase in the lung tissue from preterm fetal baboons failed to increase in response to the oxygen exposure, whereas those enzymatic activities significantly increased in lung tissue belonging to term ones.^29^

The potency of the thioredoxin system in response to oxygen exposure might be associated with some complications in preterm infants. Some studies have revealed the association between antioxidants and BPD or ROP. For instance, Fu et al. reported that a lower level of erythrocyte glutathione peroxidase is related to the development of BPD and ROP in preterm infants.^30,31^ As for non-enzymatic antioxidants, vitamin A supplementation is known to lower the risk of chronic lung disease and severe ROP.^32,33^ However, our study did not show any significant relationship between TRX-1 levels after birth and the later development of BPD and ROP among EPIs. From this study, serum TRX-1 levels cannot be used as a predictive marker for the future development of severe BPD and ROP in EPIs. Some reasons can explain this result. First, we did not assess the oxidative stress markers, such as 8-hydorxy-2´-deoxyguanosine; therefore, we could not detect whether there was an imbalance between oxidative stress and protective enzymatic activity. Second, we did not measure the levels of thioredoxin reductase or other enzymatic and non-enzymatic antioxidants. Thioredoxin reductase is important in maintaining redox homeostasis since it reduces oxidized TRX-1. Interestingly, previous studies showed that inhibiting thioredoxin reductase-1 function can attenuate lung injury by increasing the expression of glutathione.^34,35^ This finding emphasizes the complexity of the interactions among antioxidants, and thus the serum TRX-1 levels alone cannot describe the entire antioxidant system. Further studies are warranted to elucidate the relationship between the TRX system and the development of BPD and ROP in human subjects.

### Limitations

There are several limitations in our study. First, patients in our control group are a small number of neonates with TTN requiring hospitalization. Therefore, we could not establish the reference range of TRX in healthy-term neonates. It was also possible that our reference range of the TTN patients does not represent the population due to the small sample size. Second, the ranges of TRX-1 were very wide (1.07–714 ng/mL in the EPI group and 86.8–1576 ng/mL in the control group). The distribution of logit-transformed TRX-1 levels resembled normal distribution (Supplementary Fig. S1), but more samples are needed to determine its true distribution. Third, we could not deny the possibility of the influence of drugs administered to the mother on the TRX concentrations. Since ACS was reported to increase the antioxidant enzymes of superoxide dismutase, catalase, and glutathione redox cycle enzymes,^36^ we adjusted for the effect of ACS in the logistic regression models predicting severe BPD and ROP. However, since the administration of ACS, MgSO_4_, and ritodrine was significantly higher in the EPI group, these could have been related to the lower serum TRX-1 levels in that group.

## Conclusion

The serum TRX concentration on the day of birth is lower in EPIs than in infants born ≥36 weeks of gestation and hospitalized for TTN. There is no association between the serum TRX levels on admission and the subsequent development of severe BPD and ROP among EPIs.

## Supplementary information

Supplementary Information

## Data Availability

The datasets generated during and/or analyzed during the current study are available from the corresponding author on reasonable request.
