# Supplementary material for: The serum thioredoxin-1 levels are not associated with bronchopulmonary dysplasia and retinopathy of prematurity
Source: Pediatr Res. 2024 Feb 16;96(5):1275–82. doi: 10.1038/s41390-024-03078-7 (PMC11521992; doi:10.1038/s41390-024-03078-7)
Supplement: Supplementary file 1 — Supplementary Information [file 41390_2024_3078_MOESM1_ESM.pdf]

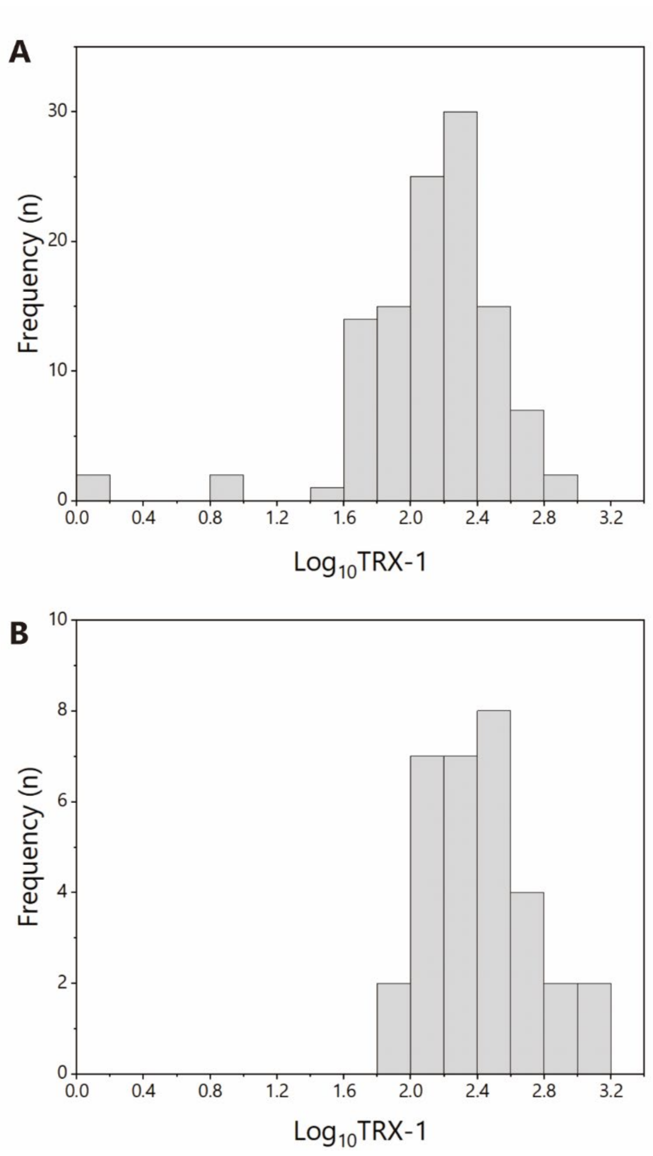

Supplementary Figure S1.

Histograms of the logit-tranformed serum thioredoxin-1 levels in extremely preterm infants (A) and controls (B). Both histograms show that the distributions of TRX-1 are almost symmetrical.

Abbreviations: TRX-1, thioredoxin-1.

Supplementary Table S1. Multivariate logistic regression analyses for severe bronchopulmonary dysplasia and severe retinopathy of prematurity with TRX-1 levels at 10–20 days of life

	Severe BPD[a]					Severe ROP[b]				
Variables	B	SE	Wald χ^2	P[c]	aOR (95% CI)	B	SE	Wald χ^2	P[c]	aOR (95% CI)
Gestational age (weeks)	−.51	.35	2.1	.15	.60 (.30–1.2)	−.51	.31	2.7	.10	.60 (.32–1.1)
Birth body weight (100 g)	−.41	.27	2.3	.13	.67 (.40–1.1)	−.021	.24	.0080	.93	.98 (.61–1.6)
Antental corticosteroids	1.3	.58	5.2	.02	14 (1.5–134)	.019	.41	.0022	.96	1.0 (.21–5.2)
TRX-1 at 10–20 days of life (10 ng/mL)	.013	.022	.36	.55	1.0 (.97–1.1)	−.014	.025	.32	.57	.99 (.94–1.0)

Area under the receiver operating characteristics curve: [a].83 and [b].76.

Abbreviations: B, coefficient; SE, standard error; aOR, adjusted odds ratio; CI, confidence interval; TRX-1, thioredoxin-1.

[c]A P value for the aOR of each variable was determined by the Wald test for the multivariate logistic regression model.

Supplementary Table S2. Multivariate logistic regression analyses for severe bronchopulmonary dysplasia and severe retinopathy of prematurity with TRX-1 levels at 36–40 weeks of postmenstrual age

	Severe BPD[a]					Severe ROP[b]				
Variables	B	SE	Wald χ^2	P[c]	aOR (95% CI)	B	SE	Wald χ^2	P[c]	aOR (95% CI)
Gestational age (weeks)	.30	.37	.65	.42	1.4 (.65–2.8)	−.53	.34	2.5	.12	.59 (.31–1.1)
Birth body weight (100 g)	−.77	.35	5.0	.03	.46 (.23–.91)	−.043	.26	.029	.87	.96 (.58–1.6)
Antenatal corticosteroids	−.39	.53	.55	.46	.46 (.058–3.6)	.27	.46	.36	.55	1.7 (.29–10)
TRX-1 at 36–40 weeks of PMA (10 ng/mL)	.21	.17	1.5	.22	1.2 (.88–1.7)	−.053	.13	.17	.68	.95 (.74–1.2)

Area under the receiver operating characteristics curve: [a].79 and [b].74.

Abbreviations: B, coefficient; SE, standard error; aOR, adjusted odds ratio; CI, confidence interval; TRX-1, thioredoxin-1.

[c]A P value for the aOR of each variable was determined by the Wald test for the multivariate logistic regression model.
